# Assessing the reporting quality of influenza outbreaks in the community

**DOI:** 10.1111/irv.12516

**Published:** 2017-11-28

**Authors:** Calvin Lo, Dominik Mertz, Mark Loeb

**Affiliations:** ^1^ Department of Pathology and Molecular Medicine McMaster University Hamilton ON Canada; ^2^ Department of Medicine McMaster University Hamilton ON Canada; ^3^ Department of Health Research Methods, Evidence and Impact McMaster University Hamilton ON Canada; ^4^ Michael G. DeGroote Institute for Infectious Diseases Research McMaster University Hamilton ON Canada

**Keywords:** influenza, outbreaks, reporting criterion, reporting quality, STROBE statement

## Abstract

**Background:**

High‐quality reporting of outbreak characteristics is fundamental to understand the behaviour of various strains of influenza virus and the impact of outbreak management strategies. However, few studies have systematically evaluated the quality of outbreak reporting.

**Objectives:**

To conduct a systematic analysis and assessment for reporting quality of influenza outbreaks based on a modified version of the STROBE statement, and to examine characteristics associated with reporting quality.

**Methods:**

A literature search was conducted across 3 online databases (PubMed, Web of Science, MEDLINE) for reports of influenza outbreaks (pandemic H1N1, avian, seasonal). The quality of reports meeting our eligibility criteria was assessed using the Modified STROBE criteria and assigned a score of 30. Mean differences (MD) and 95% confidence intervals (CI) were reported for comparisons of study characteristics.

**Results:**

Sixty‐four outbreak reports were available for analyses. The average Modified STROBE score was 20/30. Peer‐reviewed articles were associated with a better quality of reporting (MD 2.79, 95% CI 0.79‐4.78). Likewise, reports from authors affiliated with public health agencies were associated with better quality than those from academic institutions (MD 1.65, 95% CI−0.27‐3.56).

**Conclusions:**

The development of explicit reporting guidelines specifically geared towards reporting of outbreak investigations proved to be useful. Providing information on patient characteristics, investigation details in introduction and results, as well as addressing limitations that could have biased the findings, were frequently missing in the published reports.

## 1 INTRODUCTION

Influenza causes outbreaks in a broad range of settings including hospitals, schools, long‐term care centres and other confined settings.^1^ Such outbreaks are largely reported in descriptive studies such as case reports and case series, surveillance reports and cross‐sectional studies, and guidelines how these should be reported exist.^2^ Objective documentation of outbreak characteristics (eg, infected individuals, setting, duration of exposures potentially associated with outcomes) serves as the primary basis to understand epidemiological characteristics of various strains of influenza virus, and how outbreaks can potentially be managed.^3^

For influenza outbreak data to be most informative, it is important that sufficient details are reported, which may be lacking in many reports.^4^, ^5^ In fact, few studies have systematically evaluated the quality of outbreak reporting of any type of pathogen.^6^, ^7^, ^8^, ^9^ To this end, we sought to conduct a systematic analysis of the quality of reporting of influenza outbreaks and to examine characteristics associated with the quality of reporting.

## 2 METHODS

All decisions regarding eligibility criteria, search strategy, study selection, data collection, quality assessment and analysis were established a priori.

### 2.1 Eligibility criteria

We included outbreak studies involving human patients only and where the primary outbreak pathogen was either pandemic H1N1, avian or seasonal influenza. However, due to literature including zoonotic diseases (eg, avian influenza), investigations involving animal sources transmitting influenza virus to humans were eligible. We limited reports to those that at least described one or more of the following: onset of outbreak, clinical manifestations, control measures or specific diagnostic testing. Studies that evaluated surveillance systems or developed transmission models were not eligible for inclusion. We also excluded studies that did not provide a descriptive detailed account of individual outbreaks, such as annual surveillance reports.

### 2.2 Search strategy and data extraction

**Figure 1:**
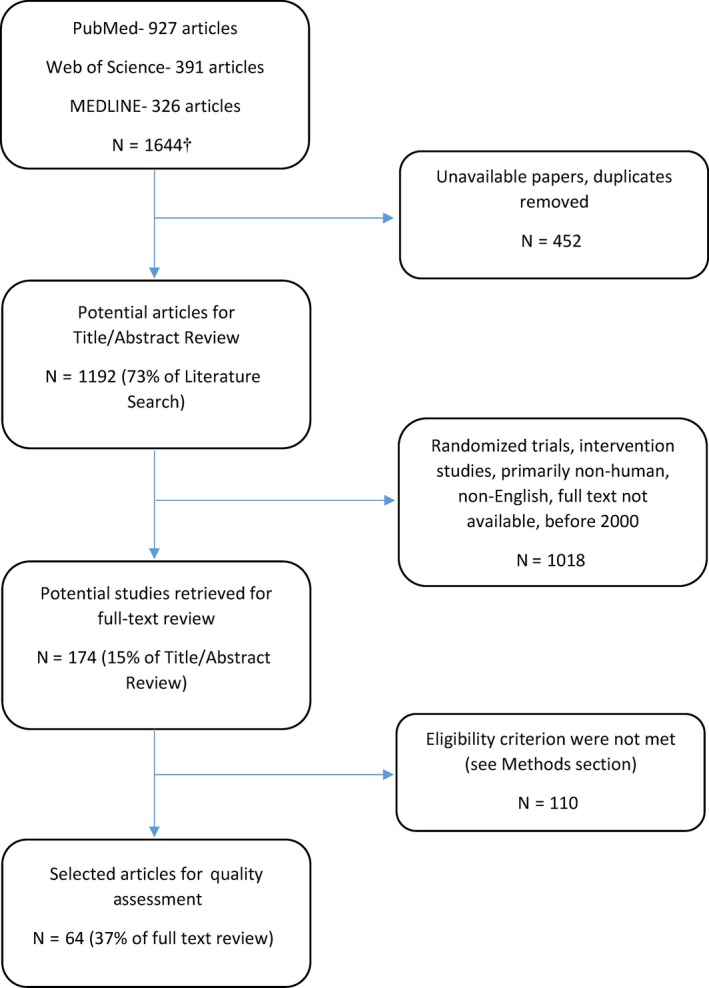
Flow diagram of outbreak reports included and excluded. ^†^ *Search terms: Outbreak* ^*^ *AND investigat* ^*^ *AND influenza* ^*^ *or flu* ^*^

We searched PubMed, Web of Science and MEDLINE for reports published from 2000 to October 2015 using a basic combination of keywords and subject headings (Figure 1 and Table S3). Our goal was not to conduct a systematic review of outbreaks but rather to obtain an unbiased sample of more recent influenza outbreaks as a general assessment of its reporting quality. *Only English language papers were included*.

### 2.3 Quality assessment

To capture the key elements of outbreak reporting and enable effective assessment of reporting quality, we made several modifications to the original STROBE (Strengthening the Reporting of Observational Studies in Epidemiology, a 22‐item checklist)^10^ resulting in a 30‐item quality assessment tool (referred to as “Modified STROBE” below; Table 1). These changes were made based on detailed discussion amongst the 3 authors. Two of the authors (DM, ML) have content as well as methodological expertise and experience applying STROBE. With each individual component of the tool worth 1 point, a quality score (“Modified STROBE score”) was computed for each outbreak report with a maximum value of 30. Individual Modified STROBE scores for each report are in Table S1, while Table S2 shows modifications made to the original assessment tool.

**Table 1:** Description of the Modified STROBE checklist and frequency of accurate reporting in the 64 outbreak reports assessed^10^, ^76^

Modified STROBE Component	Component Description	n (%) Accurately Reported
1) Title and Abstract	a) Either title, abstract or both sections clearly indicated study design.	39 (61)
	b) Study's focus and investigation details within title, abstract or both sections (eg, Influenza subtype, Geographic Location, Setting) were clearly elicited	39 (61)
	c) Informative summary provided in the abstract discussing steps taken along with investigation findings	63 (98)
2) Introduction	a) Scientific background, evidence and rationale provided for reporting and conducting investigation	64 (100)
	b) Specific objectives for study stated, include pre‐established hypotheses if applicable	57 (89)
	c) Specific quantities provided: for example number of outbreak(s)/communities reported, number of patients from influenza outbreak (suspected, confirmed, total, etc.)	64 (100)
	d) A timeline of the study was provided: includes start/finish dates of conducted investigation or outbreak	31 (48)
3) Methods	a) Present key elements of study design early in report	61 (95)
	b) Was decision to report prompted by any outcome data?	62 (97)
Outbreak characteristics	c) Number of patients admitted during outbreak	43 (67)
	d) Distributions provided for patient demographics	38 (59)
	e) Proportion admitted from other hospitals, wards, communities, etc.	26 (41)
	f) Potential risk factors for acquiring organism included	46 (72)
	g) Case definitions for outbreaks were included	54 (84)
	h) Proportions of patient outcomes were included (eg, ICU, hospitalization, mortality)	49 (77)
Outbreak location/setting	i) Description of unit, hospital, community.	29 (45)
Organization of patient and sample data	j) Provide eligibility criteria for selection of cases, participants and/or controls (more for cohort/case‐control)	54 (84)
	k) Provide number of exposed/unexposed (cohort) or controls per case (case‐control)	19 (30)
	l) Describe any efforts to address potential sources of bias	17 (27)
	m) Explain how the final study size was arrived at (or patient/case count)	62 (97)
	n) Explain how missing data were addressed	3 (5)
	o)Describe any sensitivity analysis	0 (0)
4) Results	a) Consider use of a flow diagram to depict patient or participant count at each stage of investigation	52 (81)
	b) DescriptiveGive characteristics of study participants (eg, demographic, clinical, social) + information on exposures and any other associative factors	53 (83)
	c) Timeline: charts to display duration of patient stay, date of detecting organisms, etc.	28 (44)
	d) Consideration of any confounding variables (eg, use of antibiotics, length of stay changes)	47 (73)
	e) Further results and analysesIf applicable, provided unadjusted and confounder‐adjusted estimates with confidence intervals.	(25)
5) Discussion	a) Clinical signification of observations was considered and hypotheses were reviewed in relation to the findings.	63 (98)
	b) Discuss limitations of study, accounting for any potential bias.	43 (67)
	c) Discussed generalizability (external validity) of findings and applicability with current evidence	59 (92)

### 2.4 Predictor variables

A potential association with the quality of reporting (ie, Modified STROBE score) was assessed for 7 variables: publication year, continent of outbreak, influenza strain, outbreak size, outbreak settings, author affiliation (academic institution vs non‐academic [eg, public health agencies and authorities]) and publication type (peer‐reviewed vs epidemiologic report). The predictor variables were selected a priori. We examined publications prior to and after 2009 on the basis of 2009 H1N1 pandemic given the large number of articles that followed the pandemic. Similarly, we sought to see differences by H1N1 vs seasonal or avian influenza. As STROBE (STrengthening the Reporting of OBservational studies in Epidemiology) was developed in North America and Europe, we wanted to assess differences between these and other regions. We included outbreak size to assess differences between small (local teams) vs larger outbreaks that may have national teams involved. We were interested in seeing whether hospital staff fared differently in reporting compared to the community. Along a similar line, we wanted to see whether public health officials reported better/worse compared to academics. We expected better reporting with peer‐reviewed publications so aimed to assess this as well.

Covariates with a *P*‐value of <.10 in the univariate analysis were included in the multivariate model. We chose the *P* < .10 threshold to maintain a balance between screening predictive factors for multivariate analysis but also ensuring adequate exclusion for factors deemed limited correlation with differences in quality scores. All statistical analyses were conducted using SPSS/PASW Version 18 (SPSS Inc., Chicago, IL, USA). In the event that the investigation report involved both academic and public health institutions, the corresponding author of the report was used to determine author affiliation.

## 3 RESULTS

A total of 64 of 174 (37%) studies reviewed in full text met our eligibility criteria and underwent assessment for the quality of reporting.^11^, ^12^, ^13^, ^14^, ^15^, ^16^, ^17^, ^18^, ^19^, ^20^, ^21^, ^22^, ^23^, ^24^, ^25^, ^26^, ^27^, ^28^, ^29^, ^30^, ^31^, ^32^, ^33^, ^34^, ^35^, ^36^, ^37^, ^38^, ^39^, ^40^, ^41^, ^42^, ^43^, ^44^, ^45^, ^46^, ^47^, ^48^, ^49^, ^50^, ^51^, ^52^, ^53^, ^54^, ^55^, ^56^, ^57^, ^58^, ^59^, ^60^, ^61^, ^62^, ^63^, ^64^, ^65^, ^66^, ^67^, ^68^, ^69^, ^70^, ^71^, ^72^, ^73^, ^74^

### 3.1 Quality assessment

The mean Modified STROBE score of included studies was 20.0 (standard deviation (*SD*) ± 3.6) of 30. All studies provided scientific background and context to the reported outbreak (Item 2A) and quantitative data on affected patients/reported outbreaks (2C) (Table 1). Similarly, more than 90% of the reports included an informative summary (1C), elements of study design (3A), motivations behind reporting (3B), breakdown of study size (3M), clinical significance (5A) and external validity of results (5C). In terms of poorly reported elements, the 3 items least frequently reported were 3N (addressing missing data), 3O (sensitivity analysis) and 4E (provision of risk estimates, odds ratio) at 5%, 0% and 25% of studies, respectively (Table 1). Sensitivity analyses and reporting of risk estimates were not necessarily appropriate for all studies, which explains to a large extent as to why these criteria were only met in a small minority of reports.

### 3.2 Factors associated with better reporting

Of the 64 available reports, 51 (80%) were published from 2009 and after. These reports had significantly higher Modified STROBE scores than those published prior to 2009 (MD 3.43, 95% CI 0.86 to 6.00, *P* = .010). Forty‐nine reports (77%) were peer‐reviewed publications. We found significantly higher scores with reports published in peer‐reviewed journals as opposed to public health epidemiologic reports (MD 2.79, 95% CI 0.79‐4.78, *P* = .007). The remaining 5 predictors were not found to be significantly predictive for higher Modified STROBE scores (Table 2).

**Table 2:** Predictors for reporting quality univariate and multivariate analysis

Predictor Variables	Comparison Groups	Modified STROBE Mean Score (SD)	Mean Difference (95% CI)	*P*‐values (Univariate Analysis)	*P*‐values (Multivariate Analysis)
Publication year	2009+	20.43 (3.27)	3.43 (0.86 to 6.00)	.010	.076
	−2009	17.00 (4.28)			
Journal type	Peer‐reviewed	20.65 (3.23)	2.79 (0.79 to 4.78)	.007	.034
	Epidemiologic Report	17.87 (3.85)			
Affiliation	Public Health	20.49 (3.62)	1.65 (−0.27 to 3.56)	.091	.035
	Academic Institution	18.84 (3.20)			
Outbreak size	By increase of 10 patients	20.00 (3.56)	n/a	.085	.244
Outbreak location (Continent)	North America, Europe	19.97 (3.48)	−0.06 (−1.86 to 1.73)	.945	‐
	Africa, Asia, South America, Oceania	20.03 (3.69)			
Influenza strain	H1N1	20.44 (3.13)	1.49 (−0.76 to 3.75)	.184	‐
	Avian, Seasonal	18.95 (4.33)			
Outbreak setting	Hospital	20.78 (3.26)	1.08 (−0.89 to 3.06)	.278	‐
	Community	19.70 (3.66)			

CI, confidence intervals; SD, standard deviation; ‐, not applicable.

In the multivariate model, only 4 covariates were retained as per our analysis plan: publication type, author affiliation, publication year and outbreak size. Peer‐reviewed journals (*P* = .034) remained a significant predictor for higher Modified STROBE scores. While not associated with reporting quality in univariate analysis, affiliation with public health institutions (*P* = .035) was associated with significantly higher scores in the multivariate analysis. Meanwhile, the quality of reports published after 2009 was no longer significantly better than the quality of older reports (*P* = .076).

## 4 DISCUSSION

On average, the 64 influenza outbreak reports assessed in this study met two‐thirds of the quality criteria in our Modified STROBE assessment tool. In multivariate analysis, significantly higher Modified STROBE scores were noted for peer‐reviewed articles compared to epidemiologic reports and for those written by public health‐affiliated authors compared to academic institutions.

One possible explanation for higher STROBE scores in peer‐reviewed articles was that outbreaks with significant health impact (eg, 2009 H1N1 influenza pandemic affecting several countries and spanned over months) received greater attention and were reported in higher‐impact journals that would typically put more emphasis on appropriate reporting. This is in contrast to, for example, a school‐based outbreak that spanned several days and then posted in an epidemiologic journal as a brief weekly update. *Higher STROBE scores in peer‐reviewed articles may also have been the direct result of peer review, that is, improvement in the article following the initial review*.

Reports by public health‐affiliated authors were superior in quality to those from academic institutions when included in our multivariate analysis, but not when assessed as an independent predictor. Although we referred to corresponding author's affiliation if an article had both academic and public health institutions, extensive resources for outbreak reporting and epidemiologic guidelines are likely to be consulted amongst authors regardless of institutional affiliation. With respect to our multivariate results, significantly greater scores could be related to the fact that >80% of our reports (53/64) involved help from public health authorities (local or international). Experts from these organizations are well versed in surveillance standards established by their affiliated organizations, for instance with extensive guidelines detailing the core concepts of surveillance systems and strategies to devise effective documentation of outbreak patterns.^75^

The quality of reports that have been published since 2009 was found to be higher compared to older reports in univariate, but not in the multivariate analysis. This may be related to the fact that our sample of publications since 2009 was more likely to be reported in peer‐reviewed journals (46/56 reports, 82%) and involving investigators with public health affiliation (39/56, 70%). It is likely that this was an important source of confounding which was subsequently accounted for in our multivariate analysis. Furthermore, standardized methodology for transparent reporting has been available for outbreaks report and similar epidemiologic studies (eg, STROBE, ORION)^2^, ^10^ prior to 2009.

A possible explanation for similar scores in different geographic regions could be that there were no clear differences in terms of reporting protocol across international settings. Even for outbreaks outside of North America and Europe, investigators could have originated or trained from similar public health organizations (eg, WHO, CDC) and hence proceed the investigation with limited deviations to their guidelines regardless of location. This is supported by several publications in the “non‐North American, non‐European” group which involved institutions such as WHO and CDC.^28^, ^29^, ^30^, ^34^ Despite the equal proportion of outbreak reports in North America/Europe vs other continents, non‐representative sampling may also have played a role given that “Other continents” group was mainly represented by Asian countries (69% of the 32 reports), suggesting a relative lack of publication from other continents.

Strengths of this review included a comprehensive search strategy, along with the incorporation of assessment criteria based on established grading tools for observational studies.^2^, ^10^ *We believe that our tool may be applicable to outbreaks of other respiratory viruses and pathogens*. Further applications of our modified assessment tool will help determine its generalizability to other pathogens and outbreak settings as the scope of this study was limited to influenza outbreaks that were reported in English and may not be a collective representation of outbreak populations. Furthermore, our a priori criterion for differentiating affiliation (based on first/corresponding author) may not be exact enough in defining publications where authors were affiliated with both academic and public health institutions, and hence mutual exclusivity between comparison groups could not be established; this was supported by 8 of 19 publications in our “academic institution” group.^14^, ^15^, ^16^, ^30^, ^41^, ^50^, ^51^, ^54^ The modification to the STROBE instrument was performed internally, and there was no involvement of representatives of public health agencies or any other stakeholders. This will be considered as a next step to further strengthen the instrument for future use. We also acknowledge that our modification to STROBE was based on face validity, and we did not conduct inter‐rater reliability or formal validation studies.

In conclusion, development of explicit reporting guidelines specifically geared towards publication of outbreak investigations might be useful, given there were low to moderate (<70%) rates of reports providing information on patient characteristics, investigation details in crucial parts of the report (eg, abstract/introduction, results) and in addressing limitations that could bias findings. If appropriately reported, evidence from outbreak reports can help in the management of similar outbreaks in the future and are as such an important tool for public health officials, hospital epidemiologists and other health professionals managing outbreaks.

## AUTHOR'S CONTRIBUTION

CKL, ML and DM drafted the protocol and collaboratively made modifications to the assessment tool (Modified STROBE). CKL conducted the literature search, reviewed articles for eligibility and computed individual scores for each report. All authors contributing to reviewing and finalizing specific components of the aforementioned steps. CKL conducted the data analysis and interpretation. DM and ML provided feedback on the interpretation of data. CKL, DM and ML drafted the manuscript. All authors critically reviewed the manuscript content and provided final approval of the version to publish.

## CONFLICT OF INTEREST

The authors have no competing interests.
