# Supplementary material for: Assessing the reporting quality of influenza outbreaks in the community
Source: Influenza Other Respir Viruses. 2017 Nov 28;11(6):556–63. doi: 10.1111/irv.12516 (PMC5705690; doi:10.1111/irv.12516)
Supplement: Supplementary file 1 [file IRV-11-556-s001.docx]

**Supplementary Figs 1: Distribution of Modified STROBE scores for outbreak reports (n=64)**


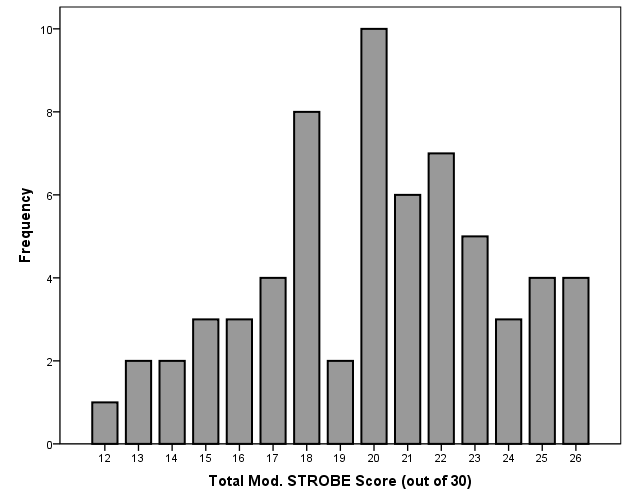


**Supplementary Table 1: Individual Modified STROBE scores for outbreak reports (n=64)^11-74^**

| **Study Name** | **Author, Year, Location** | **Modified STROBE Score (/30)** |
| --- | --- | --- |
| Outbreak of Influenza A (2009) H1N1 among Thai Healthcare Workers: Is It Time to Integrate a Vaccination Program?^11^ | Apisarnthanarak 2010, Thailand | 21 |
| Investigation of Avian Influenza (H5N1) Outbreak in Humans --- Thailand, 2004^12^ | Areechokchai et al. 2006, Thailand | 26 |
| Mass oseltamivir prophylaxis halts pandemic influenza A H1N1 2009 outbreak in a secondary school in Ashanti Region, Ghana^13^ | Asiedu-Bekoe et al. 2012, Ashanti (Ghana) | 20 |
| Influenza Outbreak Control in Confined Settings^14^ | Balicer et al. 2005, Israel (military base) | 18 |
| Multifocal Avian Influenza (H5N1) Outbreak^15^ | Balicer et al. 2007, Israel (poultry farms) | 13 |
| An outbreak of the 2009 influenza a (H1N1) virus in a children’s hospital^16^ | Bearden et al. 2012, Wisconsin (USA) | 18 |
| An outbreak of pandemic influenza A (H1N1) in Kolkata, West Bengal, India, 2010^17^ | Biswas et al. 2012, Kolkata (West Bengal, India) | 22 |
| Cluster of influenza A cases in vaccinated population of adults in Virology Laboratory in Glasgow in December 2012^18^ | Bradley-Stewart et al., 2014, Glasgow | 22 |
| Concurrent 2009 Pandemic Influenza A (H1N1) Virus Infection in Ferrets and in a Community in Pennsylvania^19^ | Campagnolo et al. 2012, Pennsylvania | 21 |
| Notes from the Field: Outbreak of Influenza A (H3N2) Virus Among Persons and Swine at a County Fair — Indiana, July 2012^20^ | CDC 2012, (Indiana, USA) | 14 |
| Influenza Outbreaks at Two Correctional Facilities — Maine, March 2011^21^ | CDC 2012, Maine (USA) | 15 |
| Severe Influenza Among Children and Young Adults with Neurologic and Neurodevelopmental Conditions^22^ | CDC 2012, Ohio (USA) | 22 |
| Outbreak of 2009 Pandemic Influenza A (H1N1) on a Peruvian Navy Ship --- June--July 2009^23^ | CDC 2010, Peruvian ship (San Francisco, California) | 20 |
| Outbreak of 2009 Pandemic Influenza A (H1N1) at a School --- Hawaii, May 2009^24^ | CDC 2010, school (Hawaii) | 20 |
| Outbreak of Swine-Origin Influenza A (H1N1) Virus Infection --- Mexico, March--April 2009^25^ | CDC 2009, Mexico (origin- Veracruz) | 20 |
| Swine-Origin Influenza A (H1N1) Virus Infections in a School --- New York City, April 2009^26^ | CDC 2009, School in New York (USA) | 17 |
| Influenza Outbreak --- Madagascar, July--August 2002^27^ | CDC 2002, Madagascar | 12 |
| Influenza B Virus Outbreak on a Cruise Ship ---Northern Europe, 2000^28^ | CDC 2001, Northern Europe cruise ship | 15 |
| Cluster of Oseltamivir-Resistant 2009 Pandemic Influenza A (H1N1) Virus Infections on a Hospital Ward among Immunocompromised Patients—North Carolina, 2009^29^ | LF Chen et al. 2009, North Carolina (USA) | 26 |
| Outbreak of H3N2 Influenza at a US Military Base in Djibouti during the H1N1 Pandemic of 2009^30^ | MT Cosby et al 2013, Djibouti | 25 |
| Transmission of Pandemic Influenza A (H1N1) Virus in a Train in China^31^ | F Cui et al. 2011, China | 24 |
| Investigation of the first cases of human-to-human infection with the new swine-origin influenza A (H1N1) virus in Canada^32^ | J Cutler et al. 2009, Nova Scotia (Canada) | 25 |
| Pre-Pandemic Outbreak of Triple-Reassortant Swine Influenza Virus Infection Among University Students, South Dakota, 2008^33^ | FS Dawood et al. 2011, South Dakota (USA) | 25 |
| Clinical aspects of influenza A (H1N1) in HIV-infected individuals in São Paulo during the pandemic of 2009^34^ | RD Bianco et al. 2010, Sao Paulo | 20 |
| Investigation of an Outbreak of Acute Respiratory Disease in Côte D’Ivoire in April 2007^35^ | E Ekaza et al. 2014, Cote D’Ivoire | 16 |
| Influenza B Outbreak on Cruise Ship off the São Paulo Coast, Brazil (2012)^36^ | EF Fernandes et al. 2014, Sao Paulo (Brazil) | 26 |
| Household Transmission of 2009 Influenza A (H1N1) Virus after a School-Based Outbreak in New York City, April–May 2009^37^ | AM France et al. 2010, New York City | 24 |
| The first cases of 2009 pandemic influenza A (H1N1) virus infection in the United States: a serologic investigation demonstrating early transmission^38^ | AM Fry et al. 2012, San Diego (California) | 22 |
| Two Clusters of Human Infection with Influenza A/H5N1 Virus in the Republic of Azerbaijan, February-March 2006^39^ | A Gilsdorf et al. 2006, Azberjian | 17 |
| Epidemiology of avian influenza A (H7N9) outbreak in Zhejiang Province, China^40^ | Z Gong et al. 2014, Zhejiang (China) | 24 |
| Outbreak of influenza virus A/H1N1 in hospital ward for immunocompromised patients^41^ | S Grund et al. 2010, Germany | 19 |
| Pandemic (H1N1) 2009 Transmission during Presymptomatic Phase, Japan^42^ | Y Gu et al. 2009, Osaka (Japan) | 16 |
| Pandemic influenza A(H1N1) 2009 outbreak in a residential school at Panchgani, Maharashtra, India^43^ | YK Gurav et al. 2009, Maharashtra (India) | 23 |
| A primary school outbreak of pandemic 2009 influenza A (H1N1) in China^44^ | Y Huai et al. 2010, various Chinese villages | 23 |
| Notes from the Field: Outbreak of 2009 Pandemic Influenza A (H1N1) Virus at a Large Public University in Delaware, April–May 2009^45^ | AD Iuliano et al. 2009, Delaware | 26 |
| Seroepidemiologic investigation of an outbreak of pandemic influenza A H1N1 2009 aboard a US Navy Vessel—San Diego, 2009^46^ | CB Khaokham et al. 2009, San Diego | 23 |
| Characteristics of Hospitalized Children with 2009 Pandemic Influenza A (H1N1): A Multicenter Study in Korea^47^ | JH Ko et al. 2012, Korea | 21 |
| Transmission of H7N7 avian influenza A virus to human beings during a large outbreak in commercial poultry farms in the Netherlands^48^ | M Koopmans et al. 2004, Netherlands | 17 |
| Outbreak of Influenza (H1N1) amongst children in a residential school^49^ | Lt Col. AS Kushwaha et al. 2012, Belgaum (India) | 22 |
| Spring 2009 H1N1 Influenza Outbreak in King County, Washington^50^ | TS Kwan-Gett et al. 2009, Washington (US) | 23 |
| Outbreak of 2009 Pandemic Influenza A (H1N1) at a New York City School^51^ | J Lessler et al. 2009, Queens (New York, USA) | 20 |
| A school outbreak of pandemic (H1N1) 2009 infection: assessment of secondary household transmission and the protective role of oseltamivir^52^ | YH Leung et al. 2010, Hong Kong | 21 |
| Epidemiological investigation of an outbreak of pandemic influenza A (H1N1) 2009 in a boarding school: Serological analysis of 1570 cases^53^ | T Li et al. 2010, Guangzhou (China) | 20 |
| Mixed Infections of Pandemic H1N1 and Seasonal H3N2 Viruses in 1 Outbreak^54^ | W Liu et al. 2010, Beijing | 13 |
| Investigation of an Outbreak of 2009 Pandemic Influenza A Virus (H1N1) Infections among Healthcare Personnel in a Chicago Hospital^55^ | SS Magill et al. 2011, Chicago | 18 |
| Pandemic Influenza A (H1N1) 2009 Outbreak Investigation in Nepal^56^ | M RK et al. 2010, Nepal | 18 |
| An Outbreak of 2009 Pandemic Influenza A (H1N1) Virus Infection in an Elementary School in Pennsylvania^57^ | TL Marchbanks et al. 2011, Pennsylvania | 21 |
| Pandemic influenza H1N1 outbreak in the Military School^58^ | J Mladenovic et al., 2013, Serbia | 18 |
| Household Transmission of Pandemic (H1N1) 2009, San Antonio, Texas, USA, April–May 2009^59^ | OW Morgan et al. 2010, Texas (USA) | 22 |
| Transmission and Effect of Multiple Clusters of Seasonal Influenza in a Swiss Geriatric Hospital^60^ | L Pagani et al. 2015, Switzerland | 18 |
| An outbreak of influenza A(H3N2) in Alappuzha district, Kerala, India, in 2011^61^ | S Peter et al. 2015, Kerala (India) | 16 |
| Nosocomial outbreak of the pandemic Influenza A (H1N1) 2009 in critical hematologic patients during seasonal influenza 2010-2011: detection of oseltamivir resistant variant viruses^62^ | CP Pollara et al. 2013, Brescia (Italy) | 15 |
| Investigation of a Pandemic H1N1 Influenza Outbreak in a Remote First Nations Community in Northern Manitoba, 2009^63^ | SL Pollock et al. 2012, Manitoba | 23 |
| Pandemic influenza A(H1N1) 2009 virus outbreak among boarding school pupils in Madagascar: compliance and adverse effects of prophylactic oseltamivir treatment^64^ | S Rajatonirina et al. 2011, Madagascar | 18 |
| Viral respiratory infections during the 2009 influenza A(H1N1) outbreak in the West Midlands Region, UK^65^ | HE Tanner et al. 2011, West Midlands Regions (UK) | 14 |
| Retrospective Investigation of an Influenza A/H1N1pdm Outbreak in an Italian Military Ship Cruising in the Mediterranean Sea, May-September 2009^66^ | M Tarabbo et al. 2011, Mediterranean (started in Italy) | 20 |
| Influenza A/H1N1/2009 outbreak in a neonatal intensive care unit^67^ | V Tsagris et al. 2012, Athens (Greece) | 17 |
| Outbreak of influenza in an overseas student travel group—Taiwan 2008^68^ | TP Tsou et al. 2010, Taiwan | 21 |
| 2009 Pandemic Influenza A (H1N1) Virus Outbreak and Response – Rwanda, October, 2009–May, 2010^69^ | J Wane et al. 2012, Rwanda | 25 |
| Novel Influenza A (H1N1) Outbreak at the U.S. Air Force Academy^70^ | CT Wiltkop et al. 2010, US Air Force (San Diego and San Antonio) | 19 |
| Possible Role of Aerosol Transmission in a Hospital Outbreak of Influenza^71^ | B CK Wong et al. 2010, Hong Kong | 20 |
| Onset and Duration of Symptoms and timing of Disease Transmission of 2009 Influenza A (H1N1) in an Outbreak in Fukuoka, Japan, June 2009^72^ | T Yamagishi et al. 2010, Fukuoka (Japan) | 22 |
| Epidemiological and Virological Characteristics of Pandemic Influenza A (H1N1) School Outbreaks in China in 2009^73^ | L Yan et al. 2012, China | 20 |
| Summer outbreak of respiratory disease in an Australian prison due to an influenza A/Fujian/411/2002(H3N2)-like virus^74^ | LC Young et al. 2005, Australia | 18 |

**Supplementary Table 2: Modifications Implemented to STROBE statement**

| **Main Components of STROBE statement** | **Modifications Made** |
| --- | --- |
| **Title and Abstract** | Included an additional component (Item 1B) which further emphasizes provision of clear and concise summaries, to help facilitate rapid collection of outbreak characteristics and investigation details (e.g., subtype, geographic location, setting, patient count). |
| **Methods** | A notable change was the removal of “eligibility criteria” for controls, as such groups do not apply in outbreak investigation. Rather, additional criteria were included with greater focus placed towards discussion of case definitions (Item 3G), description of outbreak setting (3I), distribution of patients admitted (3C), demographics (3D) and risk factors (3F) involved. Furthermore, components applicable for analytic studies (e.g., discussion on statistical methods, interactions, addressing missing data) were removed. |
| **Results** | Stronger emphasis on items 13 and 14 of the STROBE statement which now ask for the use of timelines throughout the duration of outbreak and providing patient characteristics and information on exposures and other potential confounding factors. Items from the original STROBE regarding analytic results (e.g., providing risk estimates) were merged together considering the reports were largely descriptive in nature. |
| **Discussion and Other Information** | Likewise in the “Discussion” section, a stronger emphasis was put on the clinical significance of findings, in order to bring context to outbreak severity, clinical outcomes of infected individuals, and suspected risk factors for increased transmission.  Item 22 from original STROBE was removed as source of funding was not considered relevant; our research interest was whether authors were able to effectively describe outbreak characteristics and management, and we were not interested in whether reported results might have been biased by industry involvement. |

**Supplementary Table 3: Search strategy in PubMed/Web of Science/MEDLINE, on October 24, 2015†**

| **#** | **Searches** |
| --- | --- |
| **1** | Outbreak* |
| **2** | investigat* |
| **3** | influenza* |
| **4** | flu* |
| **5** | #3 or #4 |
| **6** | #1 AND #2 AND #5 |

†As discussed in Figs. 1 and Methods Section of manuscript, search results were then screened and eliminated for duplicates across databases (or unavailable papers). After Title and Abstract Review, papers that successfully met our eligibility criterion (e.g., time range, language, patient population, not randomized trials, non-intervention studies) underwent full text review. Remaining articles underwent quality assessment with our modified tool.
